# When Does Labour Begin?

**Published:** 1902-09-06

**Authors:** 


### WHEN DOES LABOUR BEGIN?

Dr. George P. Shean [1] discusses in a very full and complete article the interesting question, When and Why does labour begin? He says that the physiology and clinical phenomena of normal pregnancy and labour are still largely misunderstood owing to the tendency of obstetric writers to accept the statements of others rather than to think for themselves. In regard to the part played by the cervix there have, he says, long been two opposing parties. According to one school the cervix shares but little in the growth of the pregnant uterus. According to the other, it not only shares in uterine growth but its canal becomes unfolded for the reception of the ovum, the upper part of the canal ultimately forming what is usually known as the lower uterine segment. Those who hold this view believe that what is usually called the internal os at the end of pregnancy, and what has been aptly termed the clinical internal os—the so-called ring of Müller—simply marks the limit of cervical unfolding. The prior view has found favour with most American writers. "In most of our text-books will be found the statement that while there occurs during pregnancy an apparent shortening of the cervix uteri this shortening is only apparent." But "it is safe to say that at present the weight of authority is in favour of the assumption that the lower uterine segment is formed either in whole or in part . . . from the upper portion of the cervical canal."

Coming now to the question, what is the condition of the cervical canal during the latter weeks of pregnancy he says that the majority of our patients are multiparæ, and that in the great majority of such cases, contrary to the opinion sometimes advanced, there is dilatation of the entire canal, including the internal os. "In nearly every case one or two fingers may be passed through the internal os to the presenting part without the slightest difficulty," and "if the ante-partum examination of pregnant women proves anything at all, it proves that in multiparæ dilatation of the cervix is no evidence of labour." Still, though the internal os is open it forms a ring—a tense and resisting ring. Dilatation of the cervical canal, including the internal os, even to the point of admitting two or more fingers may mean nothing; but dilatation of the internal os in such a way that what is left of the canal of the cervix begins to form part of the uterine cavity means labour. Only those contractions which are accompanied by this kind of dilatation deserve the name of "labour pains." At first the change can only be noted during the pains, but as labour advances it becomes appreciable during the intervals.

To sum up, he says that the statement made in many text-books that the cervix maintains its entire length during pregnancy is incorrect. The statement sometimes made that the canal of the cervix remains closed until the beginning of labour is also incorrect. Usually in multiparæ, and occasionally in primiparæ, the canal including the internal os is dilated to the extent of admitting one or two fingers two or three weeks before labour begins. Dilatation of the external or internal os or of the cervical canal is not *per se* an indication of beginning labour. Dilatation of the clinical internal os, or ring of Müller, in such a manner that it begins to form part of the uterine cavity, is at once the anatomical commencement and the diagnostic sign of true labour. Dilatation at this point is the final result of uterine distention and consequent cervical eversion. Dilatation at this point, owing to the greater resistance offered, by its effect upon the cervical ganglion, and the consequent reflex awakening of effectual uterine contractions, is the physiological cause of labour.

[1] New York Med. Rec., Aug. 23.

